# Associations Between a Genetic Liability Toward Externalizing and Behavioral Outcomes Spanning Toddlerhood Through Early Adulthood in Five Developmental Cohorts

**DOI:** 10.1016/j.jaac.2025.04.010

**Published:** 2025-04-23

**Authors:** Maia Choi, Holly E. Poore, Sarah J. Brislin, Peter B. Barr, Fazil Aliev, Stephanie Zellers, Gretchen R.B. Saunders, Jessica E. Salvatore, Scott I. Vrieze, K. Paige Harden, Abraham A. Palmer, Anu Raevuori, Antti Latvala, COGA Collaborators, Danielle M. Dick

**Affiliations:** a Rutgers University, Piscataway, New Jersey; b Veteran’s Affairs New York Harbor Healthcare System, New York, New York; c SUNY Downstate Health Sciences University, Brooklyn, New York; d University of Helsinki, Helsinki, Finland; e University of Minnesota, Minneapolis, Minnesota; f University of Texas, Austin, Texas; g University of California San Diego, San Diego, California

**Keywords:** externalizing, development, polygenic, behavioral undercontrol, substance use

## Abstract

**Objective:**

Understanding how genetic risk unfolds across development will be important for using genetics to inform prevention and early intervention. The current study leverages information from 5 large datasets to characterize behavioral manifestations of a genetic liability toward externalizing from ages 6 months to 26 years.

**Method:**

We used polygenic scores (PGS) derived from a multivariate genome-wide association study (GWAS) of externalizing that identified hundreds of significantly associated genetic variants (EXT_PGS_) to estimate associations of genetic liability with relevant phenotypes within and across developmental periods, ranging from toddlerhood to early adulthood. We used data from 5 population- and family-based datasets spanning 3 countries.

**Results:**

The EXT_PGS_ was significantly associated with a breadth of externalizing phenotypes from toddlerhood to early adulthood. Higher EXT_PGS_ was consistently associated with measures of impulsivity from early adolescence to early adulthood. Individuals with higher EXT_PGS_ were more likely to experience conduct problems and symptoms of oppositional defiant and attention-deficit/hyperactivity disorders. Furthermore, the EXT_PGS_ was associated with higher levels of substance use and problems beginning in early adolescence through early adulthood, including alcohol and illicit drug use. There was minimal evidence for sex interactions.

**Conclusion:**

A genetic liability toward externalizing is associated a wide array of behaviors and psychiatric/substance use outcomes beginning as early as childhood and through emerging adulthood. The early emergence and breadth of behaviors associated with a genetic liability toward externalizing could inform prevention and intervention.

## Plain language summary

This study leverages information from 5 large datasets to characterize behavioral manifestations of a genetic liability toward externalizing symptoms from ages 6 months to 26 years. A genetic liability toward externalizing symptoms was associated with a wide array of externalizing behaviors and psychiatric/substance use outcomes beginning as early as childhood and through emerging adulthood. The early emergence and breadth of behaviors associated with a genetic liability toward externalizing could inform prevention and intervention.

**Study registration information:**

Mapping Genetic Risk for Externalizing Behavior in Childhood, Adolescence, and Early Adulthood; https://osf.io/7g4ak/

Identifying genetic loci that contribute to psychiatric and substance use outcomes has been a major investment of the National Institutes of Health and the scientific community. Part of the rationale is the hope that genomics will advance precision medicine, potentially enabling us to move from a treatment-based model of medicine to one that is personalized, predictive, and preventative.^1^ Over the past 5 years, considerable advances have been made in our ability to successfully identify genetic loci that contribute to complex behavioral outcomes as large-scale consortia have amassed data from millions of individuals to conduct genome-wide association studies (GWAS).^2^ Results from these well-powered GWAS can be used to create polygenic scores (PGS) by summing associated variants across the genome and weighting them by their effect sizes.^2^ This enables researchers to calculate PGS in independent samples to study additional questions about the nature of genetic risk, such as how genetic risk unfolds across the lifespan, and whether the early behavioral manifestations of genetic risk differ as a function of sex, which are the areas of focus in this paper.

Understanding how genetic risk unfolds across childhood into adulthood will be critical if genomic information is to be used for prevention and early intervention. This is especially important, as most GWAS focus on adult individuals and use lifetime measures of risk. Developmental mapping of genomic risk in large datasets becomes even more important as GWAS studies adopt multivariate genomic methods. Although most initial GWAS focused on a particular disease or disorder (eg, alcohol use disorder, attention-deficit/hyperactivity disorder [ADHD], depression), genetic correlations between GWAS results for putatively distinct disorders,^3^ coupled with decades of twin research demonstrating genetic overlap across psychiatric and substance use disorders,^4^ support the idea that our current clinical diagnostic systems do not neatly reflect the nature of underlying genetic liability.^5^ For example, numerous studies have demonstrated that substance use disorders and other disorders and behaviors characterized by impulsivity and disinhibition, such as ADHD and conduct disorder, share an underlying genetic liability.^6^ Multivariate genomic methods have evolved to identify genes that operate at the level of this underlying genetic liability.^7^

The Externalizing Consortium led a multivariate GWAS with an effective sample size of 1.5 million individuals and found evidence for a common factor defined by shared genetic associations across ADHD, problematic alcohol use, cannabis initiation, age at first sexual experience, number of lifetime sexual partners, general risk tolerance, and tobacco initiation.^8^ These analyses shifted the focus away from gene identification for individual disorders to discovery for a broad, underlying liability to “externalizing,” a term used to reflect a constellation of behaviors and disorders with a core shared element reflecting behavioral disinhibition.^4,9^ This strategy enhanced power for gene discovery by capitalizing on the genetic correlations across the externalizing indicators and identified 579 genetic variants that operated through the externalizing factor. Subsequent PGS derived from this GWAS accounted for nearly 10% of the variance in externalizing outcomes in 2 independent adult samples, making it one of the most robust predictors of any behavioral outcomes to date.^8^

In the current study, we sought to extend this work by exploring the association of these PGS with externalizing behaviors across various developmental stages. To do this, we calculated PGS derived from the multivariate GWAS of Externalizing^8^ in 5 large developmental cohorts, to study associations with relevant behavioral phenotypes within and across developmental periods, ranging from toddlerhood to early adulthood. This PGS is derived from a latent genomic factor, which captures the shared genetic influences across all its indicators. Our primary aim was to characterize the behavioral manifestations of a genetic liability toward externalizing across developmental stages. Our secondary aim was to test for sex differences in how genetic liability toward externalizing unfolds across development.

## METHOD

### Samples

For all samples, analyses for this current project focused on individuals whose genomes were most similar to those from reference panels sampled from Europe, parallel to the original Externalizing GWAS, with available phenotypic and genetic data.

#### The Avon Longitudinal Study of Parents and Children (ALSPAC).

ALSPAC is a population-based, longitudinal cohort study started in 1990 in the United Kingdom.^10–12^ Pregnant women residing in Avon, UK, with expected dates of delivery between April 1, 1991, and December 31, 1992, were invited to take part in the study. The initial number of pregnancies enrolled was 14,541, and 13,988 children were alive at 1 year of age. Additional mother and child pairs that were initially eligible for the study but did not participate at birth were enrolled when the children were approximately 7 years of age; thus, the total sample size for analyses using any data collected after the age of 7 years is 15,447 pregnancies. Longitudinal data were collected on both the mother and offspring, starting when the mother was pregnant. Biological, psychological, health, environmental measures that include both parent and child report, along with genetic data, are available. Since 2014, study data have been collected and managed using REDCap electronic data capture tools hosted at the University of Bristol.^13^ The study website contains details of all of the data that are available through a fully searchable data dictionary and variable search tool (http://www.bristol.ac.uk/alspac/researchers/our-data/). Ethical approval for the study was obtained from the ALSPAC Ethics and Law Committee and the Local Research Ethics Committees. Consent for biological samples has been collected in accordance with the Human Tissue Act (2004). Analyses for this project focused on the offspring from ages 6 months to 26 years resulting in analytic numbers from 500 to 6,948.

#### The National Longitudinal Study of Adolescent to Adult Health (Add Health).

Add Health is an ongoing, nationally representative longitudinal study of US adolescents followed into adulthood that has extensive data on social, behavioral, and environmental phenotypes.^14^ Five waves of data have been collected ranging from wave one, when respondents were between 11 and 18 years (1994–1995), to wave five, when respondents were 35 to 42 years of age (2016–2018). Self-report data from waves one to four were used in the current project. Analyses for this project included unrelated individuals, resulting in analytic numbers (N) from 597 to 43,399.

#### The Collaborative Study on the Genetics of Alcoholism (COGA).

COGA is a multi-site, multi-generational, family-based study of genetic and environmental factors for alcohol use disorders. Recruitment and sample characteristics are described elsewhere.^15^ Analyses for this project include data from the prospective sample for which data collection began in 2004 through 2019, resulting in analytic numbers from 522 to 1,632.

#### The Finnish Twin Cohort Study (FinnTwin12).

FinnTwin12 is a population-based longitudinal study of Finnish twins born from 1983 to 1987.^16^ The study enrolled 5 consecutive birth cohorts from age 11 to 12 years and followed this cohort over 4 waves of data collection (11–12 years, 14 years, 17 years, and on average 22 years). The current study included all individuals from ages 13 to 26 years, resulting in analytic numbers from 849 to 1,213.

#### Minnesota Center for Twin Family Research (MCTFR).

MCTFR includes 3 longitudinal community ascertained twin cohorts. Recruitment and sample characteristics are described elsewhere.^17^ MCTFR participants have been assessed 4 to 8 times, with ages of assessment ranging from 10 to 49 years of age and with birth years ranging from 1972 to 1994. The broader MCTFR assessments focus on substance use and externalizing behaviors, with data clustered around the target ages of 11, 14, 17, 20, and 24 years. This project included all individuals from ages 11 to 26 years, resulting in analytic numbers from 558 to 2,885.

Table 1 provides descriptive characteristics of all of the samples.

### Measures

Phenotypes and the measures used to assess them at each developmental stage are described in Table 2 and Supplement 1 (available online). Broadly speaking, we used measures that assessed Personality and Behavioral Traits (eg, Big Five Personality Traits and Impulsivity), Behavior Problems (eg, Conduct Problems), and Substance Use and Problems (eg, alcohol consumption and alcohol problems).

### Statistical Analyses

Analyses were divided into developmental periods: (1) toddlerhood (0–2 years), (2) early childhood (3–5 years), (3) middle childhood (6–8 years), (4) late childhood (9–11 years), (5) early adolescence (12–14 years), (6) late adolescence (15–17 years), (7) emerging adulthood (18–21 years), (8) early adulthood (22–26 years). Analyses were performed cross-sectionally within each of these developmental periods. For repeated measures within the same developmental period, the maximum score of the measure was used.

#### Calculating Polygenic Scores.

A unified analytic pipeline was used to construct the Externalizing polygenic score (EXT_PGS_) in European-like ancestry individuals using results from the Externalizing GWAS.^8^ The pipeline relied on 2 software packages: PRS-CS,^18^ for adjusting original GWAS beta weights for linkage disequilibrium (LD), and Plink2,^19^ for constructing the EXT_PGS_ from LD-adjusted beta weights. The 1000 Genomes European reference files were used as the reference panel for estimating LD-adjusted weights in PRS-CS. Also, as the PRS-CS method is currently restricted to the ~1.3 million single-nucleotide polymorphisms (SNPs) in the high-quality consensus genotype set defined by the HapMap 3 Consortium,^20^ PGS were generated using only HapMap 3 SNPs. The original Externalizing GWAS included individuals from the COGA, AddHealth, and MCTFR samples, so when creating polygenic scores for COGA, AddHealth, and MCTFR samples we used a reduced Externalizing GWAS with individuals held out from corresponding samples to avoid upward bias in estimates. Within each sample, the PGS was z scored. Supplement 1 (available online) provides more information on quality control of genetic data and calculation of PGS.

#### Regression Models.

Regression analyses were performed separately for each developmental stage within each sample, to make use of all available data and to maximize the developmental periods covered. Each phenotype was regressed on the EXT_PGS_ and relevant covariates (the top 10 ancestry principal components, sex, and age). Robust standard errors were used to account for any multivariate non-normality, and clustered robust standard errors based on family ID were used in the family-based samples (COGA, FT12, MCTFR).

Multiple comparisons were corrected separately within each sample using the Benjamini-Hochberg procedure for controlling the false discovery rate (FDR). FDR-adjusted *p* values are reported as *p*_FDR_. In addition, given the large number and broad range of phenotypes tested, we included height as a negative control phenotype. Height was assessed by self, parent, or clinician across all samples for each available developmental period (Table 2; Supplement 1, available online).

Follow-up analyses were performed to examine interactions between the PGS and sex. For these analyses, covariates (the top 10 ancestry principal components and age) were residualized on the EXT_PGS_. Next, the phenotype was regressed on the EXT_PGS_, sex, and the EXT_PGS_ by sex interaction term. For the sex interactions, the FDR *p* value correction was performed separately for each sample.

For phenotypes that were available within the developmental period in at least 2 samples, we performed a random-effects meta-analysis of the main and interaction effect sizes separately. An FDR *p* value correction was performed across all phenotypes included in the meta-analyses. For all analyses our threshold of significance was *p*_FDR_ < .05.

Analyses were run with R version 4.1.1. This study was preregistered at on OSF (https://osf.io/7g4ak/) and deviations from the original analysis plan have been updated on OSF.

## RESULTS

**FIGURE 1:**
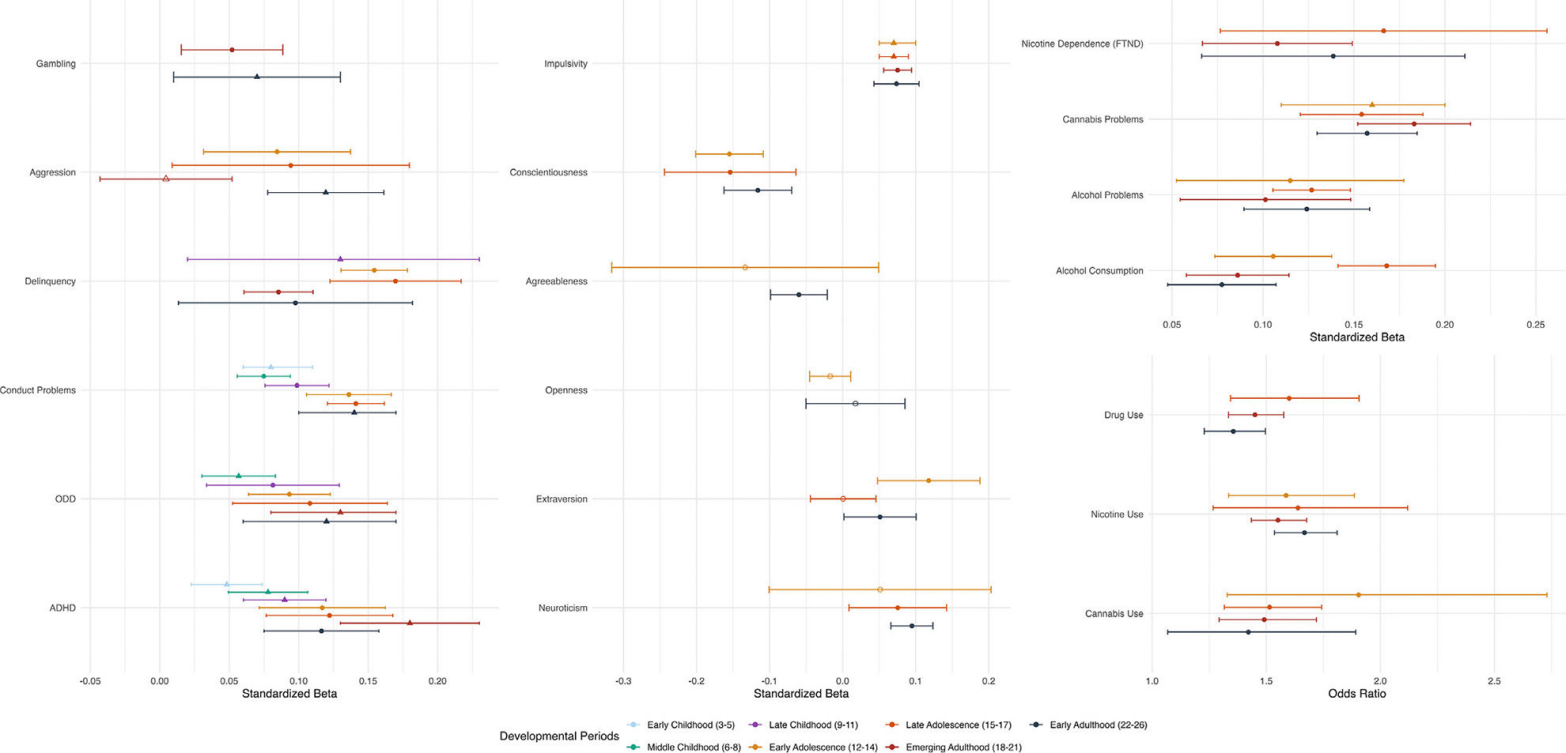
Associations Between EXT_PGS_ and Externalizing Phenotypes Across Developmental Periods **Note:** Standardized beta and odds ratio are reported for each developmental period and each phenotype measured. Circles indicate meta-analytic effects, whereas triangles indicate effects from individual studies. Filled-in shapes indicate statistically significant effects (p_FDR_ <.05), and open shapes indicate effects that were not statistically significant (p_FDR_ >.05). EXT_PGS_ = Externalizing polygenic score; OR = odds ratio. Please note color figures are available online.

Table 3 reports the meta-analysis results for phenotypes that were measured at the same developmental period in at least 2 samples. Results for the individual phenotypes, including those that were measured in only 1 sample, are separated by sample and developmental period in Supplemental Table S1a to e and Table S2a to e for the sex interactions (available online). For phenotypes that were present in at least 2 samples in 1 or more developmental periods but were available in only 1 sample at other developmental periods, we discuss individual sample and meta-analyzed effect sizes together. For example, we report all effect sizes for ADHD, which was measured in multiple samples in late adolescence and early adulthood, but only in 1 sample in middle childhood (ALSPAC) and emerging adulthood (COGA), together. Results from all available phenotypes across samples and developmental periods are reported. If a phenotype is not reported for a specific developmental period, it is because it was unavailable. Meta-analyzed effect sizes are reported as *b*_meta_, whereas sample-level effect sizes are reported as *b*_SampleName_ (eg, *b*_ALSPAC_). Table 2 includes all available measures across the samples and developmental periods. It also describes which phenotypes were included in the meta-analysis across the samples. Figure 1 displays the results of the meta-analysis.

### Personality and Behavioral Traits

The EXT_PGS_ was inconsistently associated with the Big Five personality traits. EXT_PGS_ was significantly associated with lower agreeableness in late adolescence (*b*_COGA_= −0.16) and early adulthood (*bs*_meta_ = −0.06) but not in early adolescence. The EXT_PGS_ was significantly associated with lower conscientiousness in early and late adolescence (*bs*_meta_ = −0.15) as well as early adulthood (*b*_meta_ = −0.12). The EXT_PGS_ was significantly associated with higher neuroticism in late adolescence (*b*_meta_ = −0.08) and early adulthood (*b*_meta_ = −0.09) but not in early adolescence. The EXT_PGS_ was not significantly associated with openness in early or late adolescence or in early adulthood.

The EXT_PGS_ was consistently associated with impulsivity meta-analyzed in the emerging (*β*_meta_ = 0.08) and early (*β*_meta_ = 0.07) adulthood periods.

### Behavior Problems

We observed consistent, small-to-medium and significant associations between the EXT_PGS_ phenotypes across the behavioral problems spectrum. Specifically, The EXT_PGS_ was significantly associated with high levels of conduct problems in early childhood (*b*_ALSPAC_ = 0.08), from middle childhood through late adolescence (*bs*_meta_ = 0.07–0.15), and in early adulthood (*b*_ALSPAC_ = 0.14). The EXT_PGS_ was consistently associated with measures of ADHD in early (*b*_ALSPAC_ = 0.05), middle (*b*_ALSPAC_ = 0.08), and late (*b*_ALSPAC_ = 0.08) childhood, early and late adolescence and early adulthood (*bs*_meta_ = 0.12), and in emerging adulthood (*b*_COGA_ = 0.12). The EXT_PGS_ was associated with ODD symptoms in middle childhood (*b*_ALSPAC_ = 0.06), from late childhood through late adolescence (*bs*_meta_ = 0.08–0.11), and emerging (*b*_COGA_= 0.04) and early adulthood (*b*_COGA_ = 0.05). The EXT_PGS_ was associated with delinquency in late childhood (*b*_MCTFR_ = 0.13) and from early adolescence to early adulthood (*bs*_meta_ = 0.09–0.17). The EXT_PGS_ was associated with aggression in early (*b*_meta_ = 0.08) and late (*b*_meta_ = 0.09) adolescence as well as in early (*b*_MCTFR_ = 0.12) adulthood, but the association in emerging adulthood (*b*_MCTFR_ = 0.05) was not significant. Finally, the EXT_PGS_ was significantly associated with gambling in emerging (*b*_meta_ = 0.05) and early (*b*_ALSPAC_ = 0.07) adulthood.

### Substance Use and Problems

There were consistent significant associations between EXT_PGS_ and substance use outcomes from early adolescence through early adulthood, including measures of alcohol consumption (*βs*_meta_ =0.08–0.16) and problems (*βs*_meta_ = 0.10–0.13), nicotine use (ORs_meta_= 1.55–1.67), and dependence (*βs*_meta_ = 0.11–0.17), cannabis use (ORs_meta_= 1.42–1.90) and problems (*βs*_meta_ = 0.15–0.18) and other drug use (ORs_meta_= 1.36–1.60). The EXT_PGS_ was also associated with cannabis problems in early adolescence (*β*_MCTFR_ = 0.15) and drug problems in emerging adulthood (*β*_AddHealth_ = 0.09).

### Height

There was 1 significant association between the EXT_PGS_ and height in emerging adulthood (*β*_meta_ = −0.02); all other associations with height were nonsignificant.

### Phenotypes Not Included in Meta-Analyses

#### Temperament.

In toddlers, higher EXT_PGS_ were associated with higher levels of Approach (*β*_ALSPAC_ = 0.04). There were no significant associations with the other 8 temperament subscales. In early childhood, higher EXT_PGS_ were significantly associated with higher Activity (*β*_ALSPAC_ = 0.05) and Sociability (*β*_ALSPAC_ = 0.04) and with lower Shyness (*β*_ALSPAC_ = −0.10), but not with Emotionality.

#### Additional Behavior Problems.

The EXT_PGS_ was not significantly associated Callous-Unemotionality measured in early adolescence. There were mixed associations between the EXT_PGS_ and prosocial behavior, whereby the EXT_PGS_ was significantly negatively associated with prosocial behavior in early (*β*_ALSPAC_ = −0.03) and late adolescence (*β*_ALSPAC_ = −0.06) but not significantly associated in early through late childhood and early adulthood. The EXT_PGS_ was significantly associated with antisocial behavior in middle (*β*_ALSPAC_ = 0.06) and late (*β*_ALSPAC_ = 0.18) childhood.

#### Additional Personality and Behavioral Traits.

The EXT_PGS_ was consistently significantly associated with sensation-seeking subscales of intensity measured from late childhood through emerging adulthood (*β*_ALSPAC_ = 0.13–0.20) and novelty measured in late adolescence (*β*_ALSPAC_ = 0.16) and emerging adulthood (*β*_ALSPAC_ = 0.20). The EXT_PGS_ was not significantly associated with parent or adolescent report of Social Activity, which indexed leadership, popularity, and interaction with other children, in early and late adolescence. The EXT_PGS_ was significantly negatively associated with adolescent report of compliance in early adolescence (*β*_FT12_ = −0.13), but was not associated with parent report in early adolescence or adolescent report in late adolescence.

#### Peer and Social Behaviors.

The EXT_PGS_ was significantly associated with peer problems in early adulthood (*β*_ALSPAC_ = 0.05), but not in late childhood through late adolescence.

#### Emotional Difficulties.

The EXT_PGS_ were not robustly or consistently associated with Emotional Difficulties, with significant associations observed only in middle childhood (*β*_ALSPAC_ = −0.03) and early adulthood (*β*_ALSPAC_ = .05) but in opposite directions. Associations with Emotional Difficulties were nonsignificant in the other developmental periods of early and late childhood as well as early and late adolescence.

### EXT_PGS_ by Sex Interactions

Two EXT_PGS_ by sex interactions emerged as significant: Cannabis Problems (*b*_meta_= −0.11) and Delinquency (*b*_meta_= −0.09), both in emerging adulthood. These results suggest that among female individuals, the associations between the EXT_PGS_ and cannabis problems and between EXT_PGS_ and delinquency were weaker than in male individuals. There were 4 sex interactions that were significant in the MCTFR sample. These significant interactions were conduct disorder in late adolescence (*b*_MCTFR_ = 0.002) as well as antisocial personality disorder (*b*_MCTFR_ = 0.001), cannabis dependence (*b*_MCTFR_ = 0.001), and aggression in emerging adulthood (*b*_MCTFR_ = 0.002). All these interactions were in the direction such that the association between the EXT_PGS_ and the phenotypes were stronger in female individuals compared to male individuals. However, the effect sizes for the sex interactions were very small and the *p* values were marginally significant (*p*s_FDR_ = .04).

## DISCUSSION

In this study, we used data from 5 longitudinal data sets to investigate the association of genetic risk for behavioral disinhibition with externalizing phenotypes and psychological traits analyzed cross-sectionally for developmental epochs spanning toddlerhood to early adulthood. Where possible, we meta-analyzed effect sizes for phenotypes measured in the same developmental period across multiple samples. Genetic risk reflecting behavioral disinhibition was consistently associated with phenotypes related to behavior problems (eg, ADHD and conduct problems), substance use, and sensation seeking and impulsivity traits across developmental stages. It was inconsistently associated with lower agreeableness and conscientiousness and high extraversion such that associations were significant in early adulthood, but not in early or late adolescence, and were not associated with neuroticism, emotionality, or openness. Finally, we found that the associations of genetic risk toward externalizing with cannabis problems and delinquency were weaker for female individuals compared with male individuals. Below we discuss 6 key take-aways from these analyses.

First, EXT_PGS_ were significantly associated with a broad range of behavioral traits, disorders, and substance use phenotypes. We observed the largest effect sizes for substance consumption and problems, phenotypes related to antisocial behavior, other externalizing disorders such as ADHD and oppositional defiant disorder, impulsivity, and conscientiousness. The associations with other personality traits, including extraversion, agreeableness, and neuroticism, were more modest and less consistent across samples and developmental periods.

Second, the discovery that Externalizing GWAS^8^ included indicators measured in adults, with the exception of the ADHD GWAS, which included both child and adult participants. Despite this, the EXT_PGS_ were associated with phenotypes across domains at all developmental stages. The significance and effect sizes within phenotypes (eg, impulsivity) were largely consistent across developmental periods.

Third, there was a significant association between the EXT_PGS_ and gambling in early adulthood in the 2 samples in which it was measured (ALSPAC, MCTFR). Increased vulnerability to gambling problems is particularly noteworthy, given the recent expansion of online gambling and newly permissive laws around sports betting in the United States. Although the data included in the current analyses were collected before the advent of online gambling, our results indicate that individuals who have a genetic liability toward externalizing may be particularly vulnerable to developing gambling problems.^21^

Fourth, EXT_PGS_ was not broadly associated with measures of temperament in toddlerhood, but did manifest consistent associations, including higher activity and sociability and lower shyness, in early childhood. This finding is consistent with existing literature demonstrating that stable, genetically influenced characteristics tend to emerge in early childhood.^22^

Fifth, we did not find evidence for widespread sex interactions between the EXT_PGS_ and the wide range of behavioral outcomes. Only 2 of the 62 meta-analyzed and 4 of the 347 individual sample interactions tested were significant. On the surface, this may seem inconsistent with the existing literature, which suggests that externalizing phenotypes have differential prevalence and presentations as a function of sex.^23^ Twin studies have produced inconsistent findings with respect to sex differences in genetic liability toward externalizing disorders,^23^ and GWAS have not identified sex-specific genetic influences. Our null findings may reflect true lack of differences or issues with insufficient power to detect differences with an interaction model. It is also possible that the original GWAS analyses, which used an additive model in which both sexes were analyzed together, and which were performed without an SNP by sex interaction term, contributed to the identification of SNPs that largely have similar effects across the sexes. We are currently unable to distinguish between these possibilities, but future work, in which GWAS are stratified by sex and sex-specific PGS are derived, may offer additional clarity.

Sixth, associations between EXT_PGS_ and externalizing phenotypes generally yielded small to medium effect sizes (*b*s_meta_ = 0.02–0.18). This is consistent with other PGS, and with the broader psychological literature, which typically yields small to moderate effects when considering individual outcomes.^24^ However, in previous analyses, when externalizing phenotypes were considered jointly using a phenotypic externalizing factor, the EXT_PGS_ accounted for 10% to 11% of the variance in independent samples.^8^ This indicates that the predictive power for any single externalizing outcome is smaller than the predictive power for manifesting any number of outcomes related to externalizing. This is why we believe that it is important to map the range of behavioral and psychiatric phenotypes associated with the EXT_PGS_ at various points in development.

These conclusions should be interpreted in the context of the following limitations. The current analyses included only individuals of European genomic ancestry. The Externalizing GWAS from which our PGS was derived included summary statistics from European ancestry individuals because of the limited availability of sufficiently powered GWAS in non-European ancestry samples. Given that PGS have poor portability when applied to target samples with different genetic ancestry than the original GWAS sample^25^ (eg, using a PGS developed in a European ancestry sample to an African ancestry sample), we chose to restrict our analyses. This reduces the generalizability of our findings to the global population and is a limitation more broadly of the field of genetics. It will be remedied only by large-scale efforts to collect genotypic and phenotypic data on individuals from diverse ancestry groups.^25^ A multivariate GWAS of externalizing among individuals of non-European ancestry is currently underway,^26^ which will improve our ability to create PGS for individuals of non-European ancestry in future studies.

Furthermore, observations are not independent at different developmental stages (ie, there are repeated measures from the same individuals). In addition, there were different measures across the different samples (eg, difference measures of impulsivity across the samples) along with different informants for the measures (eg, clinical interview, self-report, parent-report).

Our study included individuals from toddlerhood to early adulthood, which, while capturing a large portion of key developmental periods for externalizing, omits mid and later life stages. Extending the current analyses to later developmental periods will allow us to better understand how genetic risk manifests across the lifespan, to include relationships with physical health that may emerge later in life.

Finally, we conducted our analyses using a cross-sectional design. This allowed us to have the largest possible sample size for each phenotype, as not all individuals had data at all timepoints, and to include a wide range of phenotypes, even those assessed at only 1 developmental period. However, this approach does not allow us to model the trajectories of externalizing phenotypes across time. Future studies may incorporate longitudinal models to characterize variability of the associations between genetic risk toward externalizing and related phenotypes across time, as well as the impact that this genetic risk has on stability and change of externalizing phenotypes across development.

In conclusion, our study represents a large-scale effort to map the behavioral manifestations of a genetic liability toward externalizing, as indexed from a GWAS of 1.5 million individuals, in 5 longitudinal samples, collected across 3 countries, with broad phenotypic measurements from ages 6 months to 26 years. Our findings demonstrate the wide-ranging effects of a genetic liability toward externalizing, manifesting as increased impulsivity, elevated levels of subclinical and diagnostic behavior problems, and increased substance use experimentation and problems. Because risk behaviors associated with this genetic liability emerge in early childhood, identifying children at elevated risk early in development is possible. Previous research suggests that individuals who are most at risk are also most likely to respond to early intervention,^27^ suggesting that many of the adverse outcomes found to be associated with a genetic liability toward externalizing could be averted with prevention and early intervention efforts. For example, our team developed a new prevention program for emerging adults that provided personalized feedback on genetically influenced externalizing (eg, impulsivity, sensation seeking) and internalizing (eg, neuroticism) characteristics, to help individuals understand their risk profile and to connect them with tailored resources.^28^ Initial results suggested that this personalized program was more efficacious at reducing substance use than the standard program that directly and more narrowly targeted substance use behaviors.^29,30^ This work is currently being expanded to include feedback on EXT_PGS_, in addition to behavioral and environmental risk factors, with an RCT underway.^31^ As PGS are increasingly made available directly to the public^32^ and in clinical settings,^33^ careful characterization of the behaviors associated with genetic dispositions across development will become increasingly important to consider how to usefully and ethically harness genomic advances to improve human health and wellbeing.

## Supplementary Material

1

2

## Figures and Tables

**TABLE 1:** Sample Descriptives of Studies

Sample	Analytic N	Race/ Ethnicity	Sex	Ages	Developmental periods	N
The Avon Longitudinal Study of Parents and Children (ALSPAC)	500–6,948	White, non-Hispanic	49% female	6 mo to 26 y	Toddlerhood	N_parent_ 6,940–6,948
					Early childhood	N_parent_ 6,090–6,647
					Middle childhood	N_parent_ 4,754–5,537
					Late childhood	N_parent_ 500–6,014
					Early adolescence	N_self_ 500–6,014
						N_parent_ 3,221–5,044
					Late adolescence	N_self_ 659–5,153p
						N_parent_ 3,568–4,905
					Emerging adulthood	N_self_ 778–3,724
					Early adulthood	N_self_ 656–5,153
The National Longitudinal Study of Adolescent to Adult Health (Add Health)	597–3,399	White, non-Hispanic	53% female	12–26 y	Early adolescence	N_self_ 597–1,586
					Late adolescence	N_self_ 2,122–3,395
					Emerging adulthood	N_self_ 790–3,224
					Early adulthood	N_self_ 814–3,321
The Collaborative Study on the Genetics of Alcoholism (COGA)	522–1,632	White, non-Hispanic	51% female	12–26 y	Early adolescence	N_self_ 627–1,123
					Late adolescence	N_self_ 522–1,360
					Emerging adulthood	N_self_ 576–1,632
					Early adulthood	N_self_ 814–1,610
The Finnish Twin Cohort Study (FinnTwin12)	849–1,213	White, non-Hispanic	54% female	13–26 y	Early adolescence	N_self_ 1,156–1,213
						N_parent_ 1,135
					Late adolescence	N_self_ 855–1,092
					Early adulthood	N_self_ 849–856
Minnesota Center for Twin Family Research (MCTFR)	558–2,885	White, non-Hispanic	48% female	10–26 y	Late childhood	N_self_ 801–881
					Early adolescence	N_self_ 755–1,182
					Late adolescence	N_self_ 940–1,994
					Emerging adulthood	N_self_ 558–2,137
					Early adulthood	N_self_ 718–2,885

**Note:** All samples were limited to white/non-Hispanic participants for genetic analyses. N_self_ indicates the analytic number for self-report measures; N_Parent_ indicates the analytic number for parent report measures.

**TABLE 2:** Measures Available Across All Samples and Developmental Periods

Construct	Measure	Subscale(s)	Sample	Developmental period	Reporter
**Scales included in meta-analyses**					
Alcohol Consumption	Frequency in past 12 mo	Drink frequency, Drink quantity, Binge drinking	AddHealth	EA, LA, EmAdult, EAdult	S
	Alcohol Use Disorder Identification Test	Consumption	ALSPAC	LA, EmAdult, EAdult	S
	SSAGA	Drink frequency, Maximum drinks in 24 h	COGA	LA, EmAdult, EAdult	S
	SSAGA	Binge drinking frequency, Alcohol intoxication frequency	COGA	EmAdult, EAdult	S
	SSAGA	Drink frequency, Binge drinking frequency	FT	EA, LA, EAdult	S
	Frequency in past 12 mo	Drink frequency, Drink quantity	MCTFR	EA, LA, EmAdult, EAdult	S
	Frequency in past 12 mo	Maxium drinks in 24 h	MCTFR	LA, EmAdult, EAdult	
Alcohol Problems	AddHealth Questionnaire Battery	Alcohol problems	AddHealth	LA, EmAdult, EAdult	S
	Alcohol Use Disorder Identification Test	Problems	ALSPAC	LA, EmAdult, EAdult	S
	SSAGA	Alcohol abuse/ dependence symptoms	COGA	EA, LA, EmAdult, EAdult	S
	Structured Clinical Interview	Alcohol abuse/ dependence symptoms	MCTFR	EA, LA, EmAdult, EAdult	S
Nicotine Use	Past 30-day substance use	Cigarette	AddHealth	EA, LA, EmAdult, EAdult	S
	Past 30-day substance use	Cigarette	ALSPAC	LA, EmAdult, EAdult	S
	SSAGA	Any tobacco use in the last year	COGA	EA, LA, EmAdult, EAdult	S
	Frequency in past 12 mo	Cigarette	MCTFR	EA, LA, EmAdult, EAdult	S
Nicotine Dependence	FTND	Nicotine dependence	AddHealth	EmAdult, EAdult	S
			ALSPAC	LA, EmAdult, EAdult	S
			COGA	EmAdult, EAdult	S
			MCTFR	LA, EmAdult, EAdult	S
Cannabis Use	Past 30-day substance use	Cannabis	AddHealth	EA, LA, EmAdult, EAdult	S
	SSAGA	Cannabis use in the last year	COGA	LA, EmAdult, EAdult	S
	Frequency in past 12 mo	Cannabis	MCTFR	EA, LA, EmAdult, EAdult	S
Cannabis Problems	Cannabis Abuse Screening Test	Cannabis problems	ALSPAC	LA, EmAdult, EAdult	S
	SSAGA	Cannabis use symptoms	COGA	LA, EmAdult, EAdult	S
	Structured Clinical Interview	Cannabis use symptoms	MCTFR	EA,^a^ LA, EmAdult, EAdult	S
Other Drug Use	Past 30-day substance use	Other drugs (cocaine, opioids, stimulants, hallucinogens, inhalants)	AddHealth	EA,^a^ LA, EmAdult, EAdult	S
	Past 30-day substance use	Other drugs (cocaine, opioids, stimulants, hallucinogens, inhalants)	ALSPAC	LA, EmAdult, EAdult	S
	SSAGA	Any other drug use in the past year	COGA	LA, EmAdult, EAdult	S
Big Five Personality	IPIP	Extraversion, Agreeableness, Conscientiousness, Neuroticism, Openness	AddHealth	EAdult	S
	NEO-FFI		COGA	EA, LA, EmAdult	S
	NEO-PI-R		FT	EAdult	S
	IPIP		ALSPAC	EA	S
	MPQ	Positive Emotionality, Negative Emotionality, Constraint	MCTFR	EA, LA, EmAdult, EAdult	S
Impulsivity	AddHealth Questionnaire Battery	Impulsivity scale	AddHealth	EmAdult, EAdult	S
	UPPS	Total score	ALSPAC	EAdult	S
	BIS	Total score	COGA	EA,^a^ LA, EmAdult	S
	Karolinska Impulsivity Subscale	Impulsiveness scale	FT	LA	S
ADHD	Strengths and Difficulties Questionnaire	ADHD	ALSPAC	MC, LC, EA, LA, EAdult	S/P
	Development and Well-Being Assessment/ Structured Clinical Interview	ADHD symptoms	ALSPAC	MC, LC, EA, LA, EAdult	S
	Revised Rutter Behavior Questionnaire	Hyperactivity- Impulsivity/Inattention	ALSPAC	EC	P
	SSAGA	ADHD symptoms	COGA	EA, LA, EmAdult, EAdult	S
	SSAGA	ADHD symptoms	FT	EA	S
Conduct Problems	Strengths and Difficulties Questionnaire	Conduct symptoms	ALSPAC	MC, LC, EA, LA, EAdult	S/P
	Development and Well-Being Assessment	Conduct symptoms	ALSPAC	MC, LC, EA, LA	P
	SSAGA	Conduct symptoms	FT	EA	S
	Structured Clinical Interview	Conduct symptoms	MCTFR	LC, EA, LA	S
ODD	Development and Well-Being Assessment	ODD symptoms	ALSPAC	MC,^a^ LC, EA, LA	S/P
	SSAGA	ODD symptoms	COGA	EA, LA, EmAdult, EAdult	S
	SSAGA	ODD symptoms	FT	EA	S
	Structured Clinical Interview	ODD symptoms	MCTFR	LC, EA	S
Delinquency	Edinburgh Study for Youth Transitions and Crime Delinquency Scale	Delinquency score	ALSPAC	EA, LA, EmAdult, EAdult	S
	AddHealth Questionnaire Battery	Delinquency score	AddHealth	EA, LA, EmAdult, EAdult	S
	DBI	Delinquency score	MCTFR	LC,^a^ EA, LA	S
Aggression	MPQ	Aggression	MCTFR	EA, LA, EmAdult,^a^ EAdult^a^	S
	Multidimensional Peer Nomination Inventory	Aggression	FT	EA, LA	S/P
Gambling	Problem Gambling Severity Index	Gambling problems	ALSPAC	EmAdult, EAdult^a^	S
	Structured Clinical Interview	Gambling symptoms	MCTFR	EmAdult	S
Height	Height	Reported height	ALSPAC, AddHealth, COGA, FT	EC, LC, MC, LA, EmAdult, EAdult	S/P
	Height	Height measured in clinic	ALSPAC, MCTFR	EC, LC, MC, LA, EmAdult, EAdult	Other
**Scales not included in meta-analyses**					
Temperament	Carey Infant Toddler Scales	Activity, Rhythmicity, Approach, Adaptability, Intensity, Mood, Persistence, Distractibility, Threshold	ALSPAC	T	P
	Emotionality, Activity, and Sociability Temperament Scale	Emotionality, Activity, Shyness, Sociability	ALSPAC	EC	P
Personality and Behavioral Traits	AddHealth Questionnaire Battery	Risk Taking	AddHealth	EmAdult, EAdult	S
	Arnett Inventory of Sensation Seeking	Intensity, Novelty	ALSPAC	LC, EA, LA, EmAdult	S/P
Behavior Problems	Revised Rutter	Conduct Problems, Prosocial Behaviors	ALSPAC	EC	P
	Antisocial Behavior Questionnaire for Young Children	Total score	ALSPAC	MC, LC	S
	SSAGA	Antisocial behavior	COGA	EA, LA, EmAdult, EAdult	S
	Structured Clinical Interview	Antisocial personality disorder symptoms	MCTFR	LA, EmAdult, EAdult	S
Emotional Difficulties	Revised Rutter Parent Scale	Emotional Difficulties	ALSPAC	EC	P
	Strengths and Difficulties Questionnaire	Emotional Difficulties	ALSPAC	MC, LC, EA, LA, EAdult	S/P
Social/Peer Behavior	Strengths and Difficulties Questionnaire	Peer problems	ALSPAC	MC, LC, EA, LA, EAdult	S/P
	Multidimensional Peer Nomination Inventory	Social activity	FT	EA, LA	S/P
Other Drug Problems	AddHealth Questionnaire Battery	Drug problems	AddHealth	EmAdult, EAdult	S

Note: EA = early adolescence; EC = early childhood; EAdult = early adulthood; EmAdult = emerging adulthood; LA = late adolescence; LC = late childhood; MC = middle childhood; P = parent; S = self; T = toddlerhood.

a Denotes developmental period not included in meta-analysis.

**TABLE 3:** Results from Meta-Analysis Across All Samples and Available Phenotypes Within Developmental Period

Construct	Phenotype	Developmental period	N_meta_	Beta / OR	95% CI LL	95% CI UL	FDR-corrected *p*
Personality and Behavioral Traits	Agreeableness	Early adolescence	4,961	−0.13	−0.32	0.05	1.75E-01
		Early adulthood	3,466	−0.06	−0.10	−0.02	3.41E-03
	Conscientiousness	Early adolescence	1,382	−0.15	−0.20	−0.11	1.74E-10
		Late adolescence	6,687	−0.15	−0.24	−0.06	1.18E-03
		Early adulthood	5,519	−0.12	−0.16	−0.07	1.74E-06
	Extraversion	Early adolescence	5,039	0.12	0.05	0.19	1.41E-03
		Late adolescence	2,473	7.28E-04	−0.04	0.05	9.91E-01
		Early adulthood	4,898	0.05	1.77E-03	0.10	4.95E-02
	Neuroticism	Early adolescence	5,655	0.05	−0.10	0.20	5.43E-01
		Late adolescence	2,473	0.08	0.01	0.14	3.28E-02
		Early adulthood	5,518	0.09	0.07	0.12	3.49E-10
	Openness	Early adolescence	4,945	−0.02	−0.05	0.01	2.55E-01
		Early adulthood	2,852	0.02	−0.05	0.09	6.43E-01
	Impulsivity	Emerging adulthood	3,004	0.08	0.06	0.09	5.59E-14
		Early adulthood	6,384	0.07	0.04	0.10	5.52E-06
Behavioral Problems	Aggression	Early adolescence	1,155	0.08	0.03	0.14	2.43E-03
		Late adolescence	2,979	0.09	0.01	0.18	3.64E-02
	ADHD	Early adolescence	6,996	0.12	0.07	0.16	8.74E-07
		Late adolescence	5,220	0.12	0.08	0.17	3.27E-07
		Early adulthood	4,262	0.12	0.08	0.16	8.42E-08
	Conduct Problems	Late Childhood	6,893	0.10	0.08	0.12	2.25E-16
		Early adolescence	7,234	0.14	0.11	0.17	1.18E-17
		Late adolescence	5,569	0.14	0.12	0.16	7.79E-40
	Delinquency	Early adolescence	6,723	0.15	0.13	0.18	3.07E-35
		Late adolescence	9,942	0.17	0.12	0.22	7.58E-12
		Emerging adulthood	6,817	0.09	0.06	0.11	5.88E-11
		Early adulthood	7,045	0.10	0.01	0.18	2.86E-02
	ODD	Late Childhood	6,229	0.08	0.03	0.13	1.20E-03
		Early adolescence	7,908	0.09	0.06	0.12	1.50E-09
		Late adolescence	4,694	0.11	0.05	0.16	2.46E-04
	Gambling	Emerging adulthood	2,841	0.05	0.02	0.09	6.96E-03
Substance Use and Problems	Alcohol Consumption	Early adolescence	3,935	0.11	0.07	0.14	3.61E-10
		Late adolescence	10,912	0.17	0.14	0.19	2.19E-33
		Emerging adulthood	10,334	0.09	0.06	0.11	6.26E-09
		Early adulthood	11,108	0.08	0.05	0.11	7.06E-07
	Alcohol Problems	Early adolescence	2,963	0.11	0.05	0.18	4.88E-04
		Late adolescence	9,265	0.13	0.11	0.15	2.59E-30
		Emerging adulthood	9,540	0.10	0.05	0.15	4.03E-05
		Early adulthood	10,453	0.12	0.09	0.16	7.58E-12
	Cannabis Use (Binary)	Early adolescence	2,747	1.90	1.33	2.73	6.88E-04
		Late adolescence	6,481	1.51	1.32	1.74	1.79E-08
		Emerging adulthood	6,918	1.49	1.29	1.72	9.20E-08
		Early adulthood	7,289	1.42	1.07	1.89	2.06E-02
	Cannabis Problems	Late adolescence	4,799	0.15	0.12	0.19	2.21E-18
		Emerging adulthood	5,554	0.18	0.15	0.21	6.43E-30
		Early adulthood	6,092	0.16	0.13	0.18	3.35E-28
	Cigarette Use (Binary)	Early adolescence	3,353	1.59	1.34	1.89	3.42E-07
		Late adolescence	8,408	1.64	1.27	2.12	2.81E-04
		Emerging adulthood	8,887	1.55	1.43	1.68	2.08E-27
		Early adulthood	8,769	1.67	1.54	1.81	7.92E-33
	Fagerström Test for Nicotine Dependence	Late adolescence	1,599	0.17	0.08	0.26	4.50E-04
		Emerging adulthood	2,706	0.11	0.07	0.15	5.87E-07
		Early adulthood	3,536	0.14	0.07	0.21	2.83E-04
	Drug Use (Binary)	Late adolescence	7,058	1.60	1.34	1.91	3.25E-07
		Emerging adulthood	8,352	1.45	1.33	1.58	1.18E-17
		Early adulthood	7,214	1.36	1.23	1.50	4.38E-09
Height	Height	Late childhood	6,510	2.53E-05	−0.05	0.05	9.99E-01
		Early adolescence	9,719	0.01	−0.01	0.03	1.83E-01
		Late adolescence	12,088	−3.46E to 03	−0.02	0.01	6.71E-01
		Emerging adulthood	6,332	−0.02	−0.04	0.00	2.09E-02
		Early adulthood	5,397	−0.02	−0.04	0.01	2.20E-01

**Note:** N_meta_ indicates the combined sample size for the meta-analyzed phenotypes across available samples. Odds ratios are included for binary phenotypes. OR = odds ratio.

## Data Availability

Data are available through the individual studies.

## References

[1] Fernandes BS, Williams LM, Steiner J, Leboyer M, Carvalho AF, Berk M. The new field of ‘precision psychiatry’. BMC Medicine. 2017;15(1):80. 10.1186/s12916-017-0849-x 28403846 PMC5390384

[2] Uffelmann E, Huang QQ, Munung NS, Genome-wide association studies. Nat Rev Methods Primers. 2021;1(1):59. 10.1038/s43586-021-00056-9

[3] Martin J, Taylor MJ, Lichtenstein P. Assessing the evidence for shared genetic risks across psychiatric disorders and traits. Psychol Med. 2018;48(11):1759–1774. 10.1017/S0033291717003440 29198204 PMC6088770

[4] Krueger RF, Hicks BM, Patrick CJ, Carlson SR, Iacono WG, McGue M. Etiologic connections among substance dependence, antisocial behavior and personality: modeling the externalizing spectrum. J Abnorm Psychol. 2002;111:411–424. 10.1037/0021-843X.111.3.411 12150417

[5] Waldman ID, Poore HE, Luningham JM, Yang J. Testing structural models of psychopathology at the genomic level. World Psychiatry. 2020;19(3):350–359. 10.1002/wps.20772 32931100 PMC7491626

[6] Young SE, Friedman NP, Miyake A, Behavioral disinhibition: liability for externalizing spectrum disorders and its genetic and environmental relation to response inhibition across adolescence. J Abnorm Psychol. 2009;118(1):117. 19222319 10.1037/a0014657 PMC2775710

[7] Grotzinger AD, Rhemtulla M, de Vlaming R, Genomic structural equation modelling provides insights into the multivariate genetic architecture of complex traits. Nat Hum Behav. 2019;3(5):513–525. 30962613 10.1038/s41562-019-0566-x PMC6520146

[8] Karlsson Linner R, Mallard TT, Barr PB, Multivariate analysis of 1.5 million people identifies genetic associations with traits related to self-regulation and addiction. Nat Neurosci. 2021;24(10):1367–1376. 10.1038/s41593-021-00908-3 34446935 PMC8484054

[9] Krueger RF, Hobbs KA, Conway CC, Validity and utility of Hierarchical Taxonomy of Psychopathology (HiTOP): II. Externalizing superspectrum. World Psychiatry. 2021;20(2):171–193. 34002506 10.1002/wps.20844 PMC8129870

[10] Boyd A, Golding J, Macleod J, Cohort profile: the ‘children of the 90s’—the index offspring of the Avon Longitudinal Study of Parents and Children. Int J Epidemiol. 2013;42(1):111–127. 22507743 10.1093/ije/dys064 PMC3600618

[11] Fraser A, Macdonald-Wallis C, Tilling K, Cohort profile: the Avon Longitudinal Study of Parents and Children: ALSPAC mothers cohort. Int J Epidemiol. 2013;42(1):97–110. 10.1093/ije/dys066 22507742 PMC3600619

[12] Northstone K, Lewcock M, Groom A, The Avon Longitudinal Study of Parents and Children (ALSPAC): an update on the enrolled sample of index children in 2019. Wellcome Open Res. 2019;4(51):51. 10.12688/well-comeopenres.15132.1 31020050 PMC6464058

[13] Harris PA, Taylor R, Thielke R, Payne J, Gonzalez N, Conde JG. Research Electronic Data Capture (REDCap)—a metadata-driven methodology and workflow process for providing translational research informatics support. J Biomed Inform. 2009;42(2):377–381. 10.1016/j.jbi.2008.08.010 18929686 PMC2700030

[14] Harris KM, Halpern CT, Whitsel EA, Cohort profile: the National Longitudinal Study of Adolescent to Adult Health (Add Health). Int J Epidemiol. 2019;48(5):1415. 10.1093/ije/dyz115 31257425 PMC6857761

[15] Dick DM, Balcke E, McCutcheon V, The Collaborative Study on the Genetics of Alcoholism: sample and clinical data. Genes Brain Behav. 2023;22(5):e12860. 10.1111/gbb.12860 37581339 PMC10550787

[16] Rose RJ, Salvatore JE, Aaltonen S, FinnTwin12 cohort: an updated review. Twin Res Hum Genet. 2019;22(5):302–311. 31640839 10.1017/thg.2019.83 PMC7108792

[17] Wilson S, Haroian K, Iacono WG, Minnesota Center for Twin and Family Research. Twin Res Hum Genet. 2019;22(6):746–752. 10.1017/thg.2019.107 31796137 PMC7056536

[18] Ge T, Chen C-Y, Ni Y, Feng Y-CA, Smoller JW. Polygenic prediction via Bayesian regression and continuous shrinkage priors. Nat Commun. 2019;10(1):1776. 10.1038/s41467-019-09718-5 30992449 PMC6467998

[19] Chang CC, Chow CC, Tellier LC, Vattikuti S, Purcell SM, Lee JJ. Second-generation PLINK: rising to the challenge of larger and richer datasets. GigaScience. 2015;4(1). 10.1186/s13742-015-0047-8 PMC4342193 25722852

[20] Altshuler DM, Gibbs RA, Peltonen L, Integrating common and rare genetic variation in diverse human populations. Nature. 2010;467(7311):52–58. 10.1038/nature09298 20811451 PMC3173859

[21] Nower L, Glynn J. Adopting an affordability approach to responsible gambling and harm reduction: considerations for implementation in a North American context. Gaming Law Rev. 2022;26(9):466–476. 10.1089/glr2.2022.0020

[22] Rothbart MK. Becoming Who We Are: Temperament and Personality in Development. Guilford Press; 2011.

[23] Hicks BM, Blonigen DM, Kramer MD, Gender differences and developmental change in externalizing disorders from late adolescence to early adulthood: a longitudinal twin study. J Abnorm Psychol. 2007;116(3):433–447. 10.1037/0021-843X.116.3.433 17696699 PMC2242627

[24] Funder DC, Ozer DJ. Evaluating effect size in psychological research: sense and nonsense. Adv Methods Practices Psychol Sci. 2019;2(2):156–168. 10.1177/2515245919847202

[25] Peterson RE, Kuchenbaecker K, Walters RK, Genome-wide association studies in ancestrally diverse populations: opportunities, methods, pitfalls, and recommendations. Cell. 2019;179(3):589–603. 10.1016/j.cell.2019.08.051 31607513 PMC6939869

[26] Tanksley P, Dick DM, Harden K, Koellinger P, Karlsson LR, Williams C. Multivariate GWAS of externalizing behaviors in ~3M individuals of European and African ancestries. the Externalizing Consortium). 2023. 10.17605/OSF.IO/7PFGJ

[27] Kuo SI-C, Salvatore JE, Aliev F, Ha T, Dishion TJ, Dick DM. The family check-up intervention moderates polygenic influences on long-term alcohol outcomes: results from a randomized intervention trial. Prev Sci. 2019;20:975–985. 31175564 10.1007/s11121-019-01024-2 PMC6721991

[28] Dick DM, Saunders T, Balcke E, Genetically influenced externalizing and internalizing risk pathways as novel prevention targets. Psychol Addict Behav. 2022;36(6):595. 34110842 10.1037/adb0000759 PMC8660940

[29] Choi M, Driver MN, Balcke E, Saunders T, Langberg JM, Dick DM. Initial results from a new college substance use prevention program targeting externalizing and internalizing traits. Subst Use Misuse. 2024;59(3):421–424. 37897057 10.1080/10826084.2023.2275565 PMC10873059

[30] Choi M, Driver MN, Balcke E, Saunders T, Langberg JM, Dick DM. Bridging the gap between genetic epidemiological research and prevention: a randomized control trial of a novel personalized feedback program for alcohol and cannabis use. Drug Alcohol Depend. 2023;249:110818. 37327509 10.1016/j.drugalcdep.2023.110818 PMC10449035

[31] Choi M, Balcke E, Borle KJ, How the provision of personalized feedback about risk for addiction impacts substance use and mental health. Open Sci Frame Work. 2024.

[32] Folkersen L, Pain O, Ingason A, Werge T, Lewis CM, Austin J. Impute.me: an open-source, non-profit tool for using data from direct-to-consumer genetic testing to calculate and interpret polygenic risk scores. Front Genet. 2020;11:578. 10.3389/fgene.2020.00578 32714365 PMC7340159

[33] Wray NR, Lin T, Austin J, From basic science to clinical application of polygenic risk scores: a primer. JAMA Psychiatry. 2021;78(1):101–109. 32997097 10.1001/jamapsychiatry.2020.3049

