# Supplementary material for: Associations Between a Genetic Liability Toward Externalizing and Behavioral Outcomes Spanning Toddlerhood Through Early Adulthood in Five Developmental Cohorts
Source: J Am Acad Child Adolesc Psychiatry. Author manuscript; Available in PMC 2026 Jun 15. (PMC13267360; doi:10.1016/j.jaac.2025.04.010)
Supplement: 2 [file NIHMS2174822-supplement-2.docx]

**Supplemental Methods**

**Measures**

***ALSPAC***

**Temperament.** Temperament was assessed in toddlerhood with the Carey Infant and Toddler Scales,^1^ which include nine subscales: Activity, Rhythmicity, Adaptability, Approach, Threshold, Intensity, Mood, Distractibility, and Persistence; and in early childhood with the Emotionality, Activity, and Sociability Temperament Scale (EAS).^2^

**Personality and Behavioral Traits*.*** Personality was assessed in early adolescence with the International Personality Item Pool (IPIP),^3^ which includes the Big 5 personality dimensions, Neuroticism, Extraversion, Openness, Agreeableness, and Conscientiousness. Sensation seeking was assessed from late childhood to emerging adulthood with the Arnett Inventory for Sensation Seeking (AISS),^4^ which includes the Intensity and Novelty subscales (data from the latter of which is available only in late childhood and early adolescence). Impulsivity was assessed with the UPPS-S Impulsive Behavior Scale in early adulthood and includes five subscales: Positive Urgency, Negative Urgency, Lack of Premeditation, Lack of Perseverance, and Sensation Seeking.^5^

**Behavior Problems**. The Development and Well-being Questionnaire,^6^ which is a structured clinical interview based on *DSM-IV criteria*, was used to assess symptoms of Oppositional Defiant Disorder (ODD), Conduct Disorder (CD), and Attention-Deficit/Hyperactivity Disorder (ADHD) from middle childhood to late adolescence. ADHD was also assessed in early adulthood. In addition, non-clinical scales assessing Hyperactivity-Impulsivity/Inattention, Conduct Problems, and Prosocial Behavior from the Revised Rutter Parent Scale for Preschool Children^7^ was used in early childhood and the Strengths and Difficulties Questionnaire (SDQ)^6^ was used in middle childhood through early adulthood. Antisocial behavior was assessed in middle childhood with the Antisocial Behavior Questionnaire for Young Children^8^ and delinquency was assessed from early adolescence to early adulthood with the Edinburgh Study for Youth Transitions and Crime Delinquency Scale.^9^ Callous unemotionality was assessed in early adolescence using a six-item Callous-Unemotional scale.^10^ Gambling was assessed with the Problem Gambling Severity Index (PGSI) in emerging and early adulthood.^11^

**Emotional Difficulties.** Emotional difficulties were assessed in early childhood using the Emotional Difficulties subscales from the Revised Rutter Parent Scale for Preschool Children^7^ and from middle childhood to early adulthood using the Emotional Difficulties subscale from the SDQ.

**Social and Peer Behavior**. Peer Problems was assessed with the subscale from the SDQ^6^ from middle childhood to early adulthood.

**Substance Use.** Unless otherwise noted, substance use measures were given from late adolescence to early adulthood. Alcohol consumption and problems were assessed using the Alcohol Use Disorder Identification Test (AUDIT)^12^ and problematic cannabis use was measured with the Cannabis Use Screening Test (CAST).^13^ Participants were also asked about past 30-day nicotine and past year other drug (e.g., cocaine, opioids, stimulants) use, the latter of which was also assessed in early adolescence.

***AddHealth***

Unless otherwise noted, scales were developed for the AddHealth assessment protocol and more detailed information about their development is available elsewhere.^14^

**Personality and Behavioral Traits.** Personality was assessed in early adulthood using the Mini-International Personality Item Pool (IPIP–BF^3^)^3^ which measures the Big Five dimensions. Impulsivity was measured in emerging and early adulthood using a nine-item scale. Risk taking was assessed using a seven-item scale in emerging and early adulthood.

**Behavior Problems.** Delinquency was assessed in early adolescence, late adolescence, emerging adulthood, and early adulthood using 10-15 age-appropriate items related to violations of social norms, depending on the assessment wave.

**Substance Use.** Alcohol consumption, including past-year frequency of use, typical quantity when using, and binge-drinking frequency (5 or more consecutive drinks), was assessed in early adolescence, late adolescence, emerging adulthood, and early adulthood. Alcohol problems, which includes questions related to impairment in relationships and health due to drinking, was assessed in late adolescence, emerging adulthood, and early adulthood. Past 30-day nicotine use was measured from early adolescence to early adulthood and problematic nicotine use, assessed using Fagerstrom Test for Nicotine Dependence (FTND)^15^ in emerging and early adulthood. Past 30-day cannabis and past 30-day other drug use were assessed from early adolescence to early adulthood. Finally, problematic drug use was assessed using six-items related to impairment in functioning caused by use in emerging and early adulthood.

***COGA***

**Personality and Behavioral Traits.** Personality was assessed using the Five Factor Personality Inventory (NEO-FFI),^16^ which measures the Big Five dimensions, from early adolescence to emerging adulthood. Impulsivity was assessed using the Barrett Impulsiveness Scale (BIS),^17^ which measures three facets of impulsivity: Cognitive, Motor, and Nonplanning.^17^

**Behavior Problems.** Individuals younger than age 18 at the time of the interview completed the Child Semi-Structured Assessment for the Genetics of Alcoholism (C-SSAGA), an interview for children and adolescents based on the SSAGA and developed for COGA.^18^ Individuals 18 years and older at the time of the interview completed the SSAGA, which has been found to produce reliable and valid DSM-based criterion counts.^18^ DSM-IV maximum lifetime symptom counts of ADHD and ODD, as well as a broad range of antisocial behaviors, were assessed from early adolescence to early adulthood.

**Substance Use.** Substance use outcomes were also assessed using the SSAGA and C-SSAGA.^18^ Alcohol consumption, including past-year frequency of use and maximum drinks in a 24-hour period, was assessed from late adolescence to early adulthood. Additional alcohol consumption behaviors, including frequency of intoxication and frequency of binge drinking (5 or more consecutive drinks) were assessed in emerging and early adulthood. DSM-IV alcohol dependence symptoms were assessed from late adolescence to early adulthood. Past-year cannabis and other drug use were assessed from late adolescence to early adulthood, and past-year nicotine use was assessed in these stages as well as early adolescence. DSM-IV cannabis dependence symptoms were assessed from late adolescence to early adulthood and problematic nicotine use was assessed using the FTND^15^ in emerging and early adulthood.

***FinnTwin12***

**Personality and Behavioral Traits**. Personality was assessed using the NEO Personality Inventory (NEO-PI-R),^19^ which measured the Big Five dimensions, in early adulthood. Impulsivity was assessed with the Karolinska Impulsivity Scale in late adolescence. The Multidimensional Peer Nomination Inventory (MPNI)^20^ adjustment scale assessed compliance, as reported separately by self and parents in early and late adolescence.

**Behavior Problems.** DSM-IV maximum lifetime symptom counts of ADHD, ODD, and CD were assessed in early adolescence using the C-SSAGA and SSAGA,^18^ described above. Parent and self-report of aggression were assessed with the MPNI^20^ in early and late adolescence.

**Social and Peer Behaviors.** The MPNI^20^ also assessed parent and self-report of social activity (e.g., leadership, popularity, and interactions with peers) in early and late adolescence.

**Substance Use.** Substance use was assessed using the C-SSAGA and SSAGA.^18^ Alcohol consumption, including frequency of use and f intoxication, was assessed from early adolescence to early adulthood. DSM-IV symptoms of alcohol dependence were assessed in early adolescence and early adulthood.

***MCTFR***

**Personality and Behavioral Traits.** Personality was assessed using the Multidimensional Personality Questionnaire (MPQ),^21^ which includes positive affect, negative affect, and constraint dimensions, from early adolescence to early adulthood. These domains map onto extraversion, neuroticism, and conscientiousness, respectively, in the Big Five.

**Behavior Problems.** DSM-III-R symptom counts of childhood disorders were assessed using the SCID-IV.^22^ Symptoms of ODD were assessed in late childhood and early adolescence, and CD in late childhood, early adolescence, and late adolescence, ASPD in late adolescence, emerging adulthood, and early adulthood. Finally, DSM-IV symptoms of gambling disorder were assessed in emerging adulthood. Delinquency was assessed using the Delinquent Behavior Inventory^23^ from late childhood to late adolescence.

**Substance Use.** Unless otherwise noted, all substance use variables were assessed in early adolescence, late adolescence, emerging adulthood, and early adulthood. Alcohol consumption was measured by assessing frequency of use, average quantity consumed when using, and maximum drinks in a 24-hour period, the latter of which was assessed only from late adolescence to early adulthood. Participants reported the frequency of nicotine and cannabis use. Alcohol and cannabis abuse and dependence symptoms were assessed using DSM-III-R criteria^24^ Finally, problematic nicotine use was assessed using the FTND^15^ from late adolescence to early adulthood.

***Quality Control of Genetic Data***

Before performing genetic analyses, our study team first filtered the data with genotyping rate > 95%, MAF > 0.01 and H-W p-value > 10^-6^. Next, this filtered data was imputed with 1000 genomes phase 3 European reference panel. After imputation we filtered again with the same genotyping rate > 95%, MAF > 0.01 and H-W p-value > 10^-6^.

***Calculating Polygenic Scores***

A unified analytic pipeline was used to construct the Externalizing polygenic score (EXT_PGS_) in European-like ancestry individuals using results from the Externalizing GWAS.^25^ The pipeline relied on two software packages: PRS-CS,^26^ for adjusting original GWAS beta weights for linkage disequilibrium (LD), and Plink2,^27^ for constructing the EXT_PGS_ from LD-adjusted beta weights. The 1000 Genomes European reference files were used as the reference panel for estimating LD-adjusted weights in PRS-CS. Also, as the PRS-CS method is currently restricted to the ~1.3 million SNPs in the high-quality consensus genotype set defined by the HapMap 3 Consortium;^28^ polygenic scores were generated only using HapMap 3 SNPs. The original Externalizing GWAS included individuals from the MCTFR samples, so when creating polygenic scores for this sample we used a reduced Externalizing GWAS with individuals in MCTFR held out to avoid upward bias in estimates. Within each sample, the PGS scores z scored. PRS-CS was run separately for each sample.

The number of available SNPs varied across data sets and within some data sets, in which the number of SNPs per individual varied based on missingness. For each sample the number of SNPs were 947050 to 954220 for ALSPAC, 969262 for AddHealth, 741934 to 968026 for COGA, 963561 for FT, 971740 for MCTFR. Since the SNPs available varied across the samples, PRS-CS was calculated separately in each sample with all available SNPs. This was done to maximize the number of SNPs used and prevent loss of information by restricting to the common SNPs across all samples.

***Regression Models***

The age associated with the max score within each developmental period was used as a covariate. If the max score of a measure occurred more than once in the same developmental period, the oldest age associated with that score was used as the covariate. Linear regression fitted with ordinary least squares was used for continuous outcomes and logistic regression was used for binary outcomes.

For the regression models, the variance explained by a baseline model including only covariates was compared to a model including covariates and the PGS. The proportion of variance, ΔR^2^(Nagelkerke pseudo R^2^ for logistic models), explained by the PGS was calculated by subtracting the R^2^ value of the baseline model from the R^2^ value of the model including the PGS. ΔR^2^ estimates were bootstrapped using 1000 samples to create 95% confidence intervals.

Analyses were run with R version 4.1.1. Packages used were *dplyr*,^29^ *Sandwich,^30^* *lmTest*,^31^ *meta*,^32^ rstaix,^33^ and *performance*.^34^

***FDR P value corrections***

Multiple comparisons were corrected using the Benjamini-Hochberg procedure for controlling the False Discovery Rate (FDR). For both aims, we performed the p-value correction separately for each sample. FDR adjusted *p*-values are reported as *p*_FDR_. For all FDR p value corrections, an alpha of .05 for each q-value (i.e., FDR corrected p value).

References

1. Carey WB, McDevitt SC. Revision of the infant temperament questionnaire. *Pediatrics*. 1978;61(5):735-739.

2. Buss AH, Plomin R. *Temperament (PLE: Emotion): Early developing personality traits*. Psychology Press; 2014.

3. Goldberg LR. International Personality Item Pool: A scientific collaboratory for the development of advanced measures of personality and other individual differences. 1999.

4. Arnett J. Sensation seeking: A new conceptualization and a new scale. *Personality and individual differences*. 1994;16(2):289-296.

5. Cyders MA, Littlefield AK, Coffey S, Karyadi KA. Examination of a short English version of the UPPS-P Impulsive Behavior Scale. *Addict Behav*. Sep 2014;39(9):1372-6. doi:10.1016/j.addbeh.2014.02.013

6. Goodman R. Psychometric properties of the strengths and difficulties questionnaire. *Journal of the American Academy of Child & Adolescent Psychiatry*. 2001;40(11):1337-1345.

7. Elander J, Rutter M. Use and development of the Rutter parents' and teachers' scales. *International Journal of Methods in Psychiatric Research*. 1996;

8. Loeber R, Stouthamer-Loeber M, Van Kammen WB, Farrington DP. Development of a new measure of self-reported antisocial behavior for young children: Prevalence and reliability. *Cross-national research in self-reported crime and delinquency*. 1989:203-225.

9. Smith DJ, McVie S. Theory and Method in the Edinburgh Study of Youth Transitions and Crime. *The British Journal of Criminology*. 2003;43(1):169-195. doi:10.1093/bjc/43.1.169

10. Moran P, Ford T, Butler G, Goodman R. Callous and unemotional traits in children and adolescents living in Great Britain. *The British Journal of Psychiatry*. 2008;192(1):65-66.

11. Holtgraves T. Evaluating the problem gambling severity index. *Journal of gambling studies*. 2009;25:105-120.

12. Bohn MJ, Babor TF, Kranzler HR. The Alcohol Use Disorders Identification Test (AUDIT): validation of a screening instrument for use in medical settings. *Journal of Studies on Alcohol*. 1995;56(4):423-432. doi:10.15288/jsa.1995.56.423

13. Sznitman SR. The Cannabis Abuse Screening Test (CAST) revisited: examining measurement invariance by age. *Int J Methods Psychiatr Res*. Dec 2017;26(4)doi:10.1002/mpr.1529

14. Harris KM, Halpern CT, Whitsel EA, et al. Cohort Profile: The National Longitudinal Study of Adolescent to Adult Health (Add Health). *International Journal of Epidemiology*. 2019;48(5):1415-1415k. doi:10.1093/ije/dyz115

15. Heatherton TF, Kozlowski LT, Frecker RC, Fagerström KO. The Fagerström Test for Nicotine Dependence: a revision of the Fagerström Tolerance Questionnaire. *Br J Addict*. Sep 1991;86(9):1119-27. doi:10.1111/j.1360-0443.1991.tb01879.x

16. Costa PT, McCrae RR. *NEO PI/FFI manual supplement for use with the NEO Personality Inventory and the NEO Five-Factor Inventory*. Psychological Assessment Resources; 1989.

17. Patton JH, Stanford MS, Barratt ES. Factor structure of the barratt impulsiveness scale. *Journal of Clinical Psychology*. 1995;51(6):768-774. doi:<https://doi.org/10.1002/1097-4679(199511)51:6><768::AID-JCLP2270510607>3.0.CO;2-1

18. Bucholz KK, Cadoret R, Cloninger CR, et al. A new, semi-structured psychiatric interview for use in genetic linkage studies: a report on the reliability of the SSAGA. *Journal of Studies on Alcohol*. 1994;55(2):149-158. doi:10.15288/jsa.1994.55.149

19. Costa PT, McCrae RR. *Neo personality inventory-revised (NEO PI-R)*. Psychological Assessment Resources Odessa, FL; 1992.

20. Pulkkinen L, Kaprio J, Rose RJ. Peers, teachers and parents as assessors of the behavioural and emotional problems of twins and their adjustment: the Multidimensional Peer Nomination Inventory. *Twin Research*. 1999;2(4):274-285. doi:10.1375/twin.2.4.274

21. Tellegen A, Waller NG. Exploring personality through test construction: Development of the Multidimensional Personality Questionnaire. *The SAGE handbook of personality theory and assessment*. 2008;2:261-292.

22. Glasofer DR, Brown AJ, Riegel M. Structured clinical interview for DSM-IV (SCID). *Encyclopedia of feeding and eating disorders*. 2015:1-4.

23. GIBSON HB. Self-reported Delinquency among Schoolboys, and their Attitudes to the Police. *British Journal of Social and Clinical Psychology*. 1967;6(3):168-173. doi:<https://doi.org/10.1111/j.2044-8260.1967.tb00517.x>

24. Wittchen HU. Reliability and validity studies of the WHO--Composite International Diagnostic Interview (CIDI): a critical review. *J Psychiatr Res*. Jan-Feb 1994;28(1):57-84. doi:10.1016/0022-3956(94)90036-1

25. Karlsson Linner R, Mallard TT, Barr PB, et al. Multivariate analysis of 1.5 million people identifies genetic associations with traits related to self-regulation and addiction. *Nat Neurosci*. Oct 2021;24(10):1367-1376. doi:10.1038/s41593-021-00908-3

26. Ge T, Chen C-Y, Ni Y, Feng Y-CA, Smoller JW. Polygenic prediction via Bayesian regression and continuous shrinkage priors. *Nature Communications*. 2019/04/16 2019;10(1):1776. doi:10.1038/s41467-019-09718-5

27. Chang CC, Chow CC, Tellier LC, Vattikuti S, Purcell SM, Lee JJ. Second-generation PLINK: rising to the challenge of larger and richer datasets. *GigaScience*. 2015;4(1)doi:10.1186/s13742-015-0047-8

28. Altshuler DM, Gibbs RA, Peltonen L, et al. Integrating common and rare genetic variation in diverse human populations. *Nature*. Sep 2 2010;467(7311):52-8. doi:10.1038/nature09298

29. Wickham H, Francois R, Henry L, Müller K. dplyr. *A Grammar of Data Manipulation 2020 [Last accessed on 2020 Aug 12] Available from*. 2014:Rproject.

30. Zeileis A, Lumley T, Berger S, Graham N, Zeileis MA. Package ‘sandwich’. *R package version*. 2019:2.5-1.

31. Hothorn T, Zeileis A, Farebrother RW, et al. Package ‘lmtest’. *Testing linear regression models* [*https://cran*](https://cran) *r-project org/web/packages/lmtest/lmtest pdf Accessed*. 2015;6

32. Schwarzer G. meta: An R package for meta-analysis. *R news*. 2007;7(3):40-45.

33. Kassambara A. rstatix: Pipe-friendly framework for basic statistical tests. *CRAN: Contributed Packages*. 2019;

34. Lüdecke D, Ben-Shachar MS, Patil I, Waggoner P, Makowski D. performance: An R package for assessment, comparison and testing of statistical models. *Journal of Open Source Software*. 2021;6(60)
